# Some living eukaryotes during and after scanning electron microscopy

**DOI:** 10.1186/s42649-021-00065-8

**Published:** 2021-11-04

**Authors:** Ki Woo Kim

**Affiliations:** Department of Ecology and Environmental System, Kyungpook National University, 37224 Sangju, Republic of Korea

**Keywords:** Desiccation, Radiation, Scanning electron microscopy, Vacuum

## Abstract

Electron microscopy (EM) is an essential imaging method in biological sciences. Since biological specimens are exposed to radiation and vacuum conditions during EM observations, they die due to chemical bond breakage and desiccation. However, some organisms belonging to the taxa of bacteria, fungi, plants, and animals (including beetles, ticks, and tardigrades) have been reported to survive hostile scanning EM (SEM) conditions since the onset of EM. The surviving organisms were observed (i) without chemical fixation, (ii) after mounting to a precooled cold stage, (iii) using cryo-SEM, or (iv) after coating with a thin polymer layer, respectively. Combined use of these techniques may provide a better condition for preservation and live imaging of multicellular organisms for a long time beyond live-cell EM.

## Introduction

Electron microscopy (EM) is an essential imaging method in biological sciences. Due to its higher spatial resolution than light microscopy, EM provides unparalleled information on external and internal structures, allowing in-depth documentation of biological processes. This remarkable nature of EM stems from the high-energy electron beam in a high-vacuum environment that prevents electron scattering (Nishiyama et al. 2010). However, the exposure of biological specimens to the energetic electron beam can lead to radiation damage due to the irreversible breakage of molecular bonds and generation of reactive chemical species (de Jonge and Peckys 2016). Since soft materials, including cells, viruses, and macromolecules, are made up of low atomic number elements, they get structurally damaged due to electron beams (He et al. 2019). Noninvasive interaction-free quantum measurements were also proposed to reduce specimen damage in EM while achieving molecular-level resolution (Putnam and Yanik 2009). Search for a proper condition to visualize living organisms has been advanced through low-dose imaging and liquid-phase EM (Kennedy et al. 2017; Wu et al. 2020).

High-vacuum conditions used during EM (approximately up to 10^−7^ Pa) is another limiting factor for the survival of biological specimens. These conditions cause rapid evaporation of water from the organisms, resulting in their collapse and cell death (Takaku et al. 2013). Thus, imaging biological specimens in their native state at the onset of EM is challenging. To avoid specimen damage during conventional scanning EM (SEM), several preparations are necessary before SEM observations. Wet biological specimens are commonly subjected to fixation, dehydration, drying, and conductive coating.

Despite such constraints on biological specimens, there have been reports on living eukaryotic organisms surviving hostile SEM environments. Considering that SEM uses a relatively low-energy electron beam for imaging, radiation damage can be reduced. Some organisms survive the near-absence of water by entering a quiescent state and later resuming activity upon rehydration (Hibshman et al. 2020). The main criterion for animal survival was their capability to move and develop into the next growth stage after being removed from SEM. Plant specimens are measured for their cytoplasmic activity after SEM observations. After SEM observation with graphene veils, bacterial growth was confirmed through subsequent incubation (Koo et al. 2020). In this study, I reviewed eukaryotes surviving SEM observations. This review can provide clues to understand the survival of organisms and offer a basis for live-organism EM beyond live-cell EM.

## Examples of organismal growth after SEM observation

### Beetles surviving vacuum and electron beam radiation

Insects observed using SEM without chemical fixation were first recognized to be alive after SEM observation. The confused flour beetle, *Tribolium confusum*, is the first identified organism that survived SEM’s vacuum and electron beam radiation (Pease et al. 1966). The eggs, larvae, pupae, and adults of the beetle were kept in the SEM chamber (10^−1^ Pa) for 30 min. Furthermore, other specimens of the same developmental stages were observed using SEM at 25 kV for at least 2 min to 1 h. The specimens were assessed and considered to have survived the conditions of SEM if they passed into the next developmental stage with normal morphology.

**Fig. 1:**
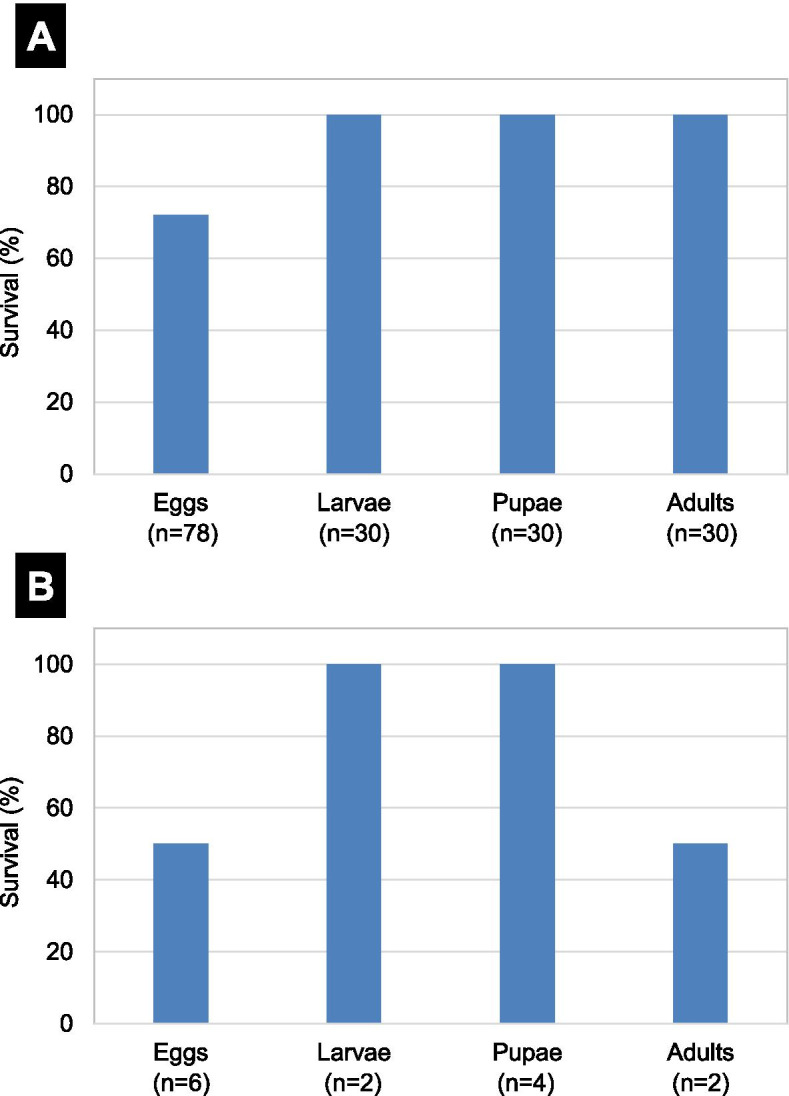
Survival percentage of the confused flour beetle *Tribolium confusum*. **A** Survival after exposure to a pressure of 10^−1^ Pa in the SEM chamber for 30 min. **B** Survival after exposure to SEM observations for at least 2 min to 1 h (Pease et al. 1966)

*T. confusum* survived the vacuum condition in all developmental stages (Fig. 1 A) (Pease et al. 1966). However, a decrease in survival percentages (50 %-100 %) was found after SEM observation (Fig. 1B). Furthermore, the lifetime of the surviving adult was significantly shortened based on a 2-month-long observation after SEM. These results indicate that most of the specimens resumed their activity and underwent complete metamorphosis.

### Stamen hair cells surviving the cold stage of low-temperature SEM

**Fig. 2:**
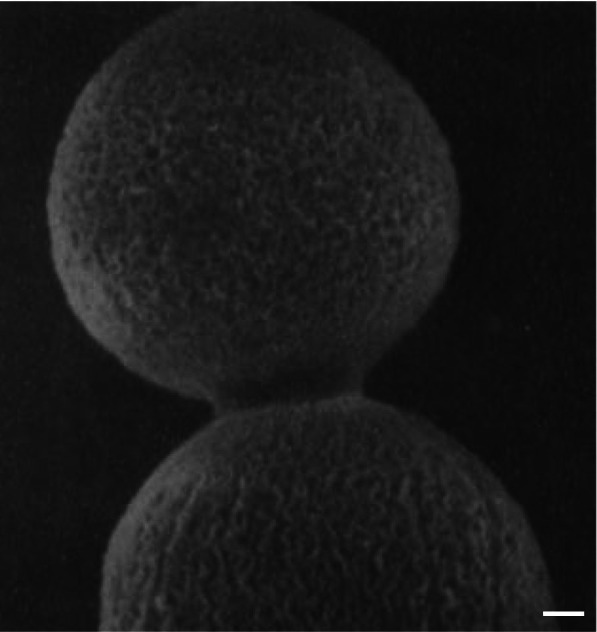
Low-temperature scanning electron micrograph of stamen hair cells of the herbaceous perennial plant *Tradescantia reflexa*. The specimen was mounted on a cryo-stage, cooled to -15℃, and observed using SEM at 10 kV for less than 10 min. Bar = 5 μm (Kaneko et al. 1985 with permission from the publisher)

Although many attempts have been made to resume animal growth after SEM observations, only few attempts have been made for plant specimens. As a criterion for survival, the rates of protoplasmic streaming were measured using an optical microscope after the removal of plant specimens from SEM (Kaneko et al. 1985). Young stamen hair cells of a perennial herbaceous plant, *Tradescantia reflexa*, were mounted on a cold stage cooled to -15℃ and observed using SEM at 10 kV for less than 10 min (Fig. 2). The rates of protoplasmic streaming were almost the same as those of nonirradiated control cells (5.5 μm/s at 20℃).

The low-temperature SEM method using a cold stage at -15℃ was distinguished from cryo-SEM because the specimens were not likely to be frozen during quick observation and photography (Kaneko et al. 1985). Such a difference was considered to make the specimens alive during SEM observation. However, no further evidence was presented as to how long the stamen hair cells survived or whether they continued to grow and differentiate into next developmental stages (Read and Lord 1991).

### Fungal spores surviving cryo-fixation or chemical fixation protocols

Ascospores of a filamentous, dung-colonizing fungus, *Sordaria macrospora*, were poured onto cellophane squares for at least 1 h (Read and Lord 1991). The resultant spore monolayers were mounted on stubs, subjected to various cryo-fixation or chemical fixation regimes, and observed using SEM. Following examination at 5 and 40 kV for 5 min, the cellophane squares were removed from the stubs, placed on a culture medium, and incubated at 27℃ for 48 h. These preparations were a requisite for tracing individual ascospore behavior after SEM observations.

**Fig. 3:**
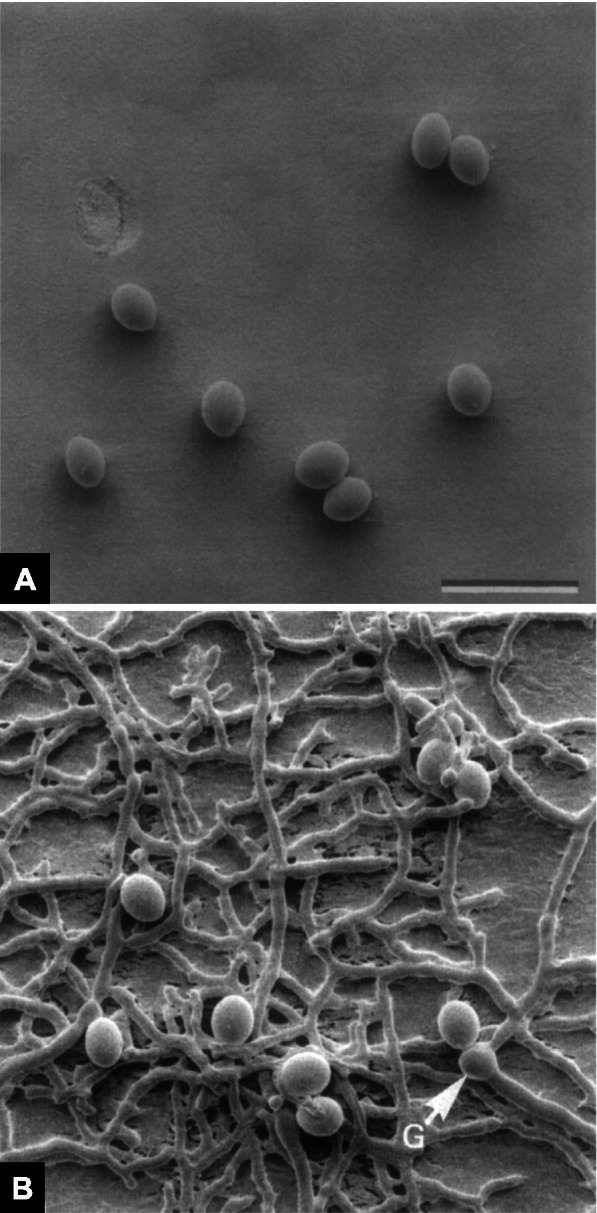
Cryo-scanning electron micrographs of the same ascospores of a fungus *Sordaria macrospora*. **A** Before germination. Bar = 50 μm. **B** After germination. G = germination vesicle (Read and Lord 1991 with permission from the publisher)

Before germination, the lemon-shaped ascospores were attached to the cellophane square (Fig. 3 A). Even after SEM observations, most ascospores (79 %-99 %) survived the cryo-fixation regimes, germinated, and produced ramified mycelia (Fig. 3B) (Read and Lord 1991). Also, a germination vesicle emerged from the spore germ pore. The germinated ascospores later formed sexual reproductive structures (Read and Lord 1991). Meanwhile, it was observed that the chemical fixation regimes reduced the germination of ascospores (0 %-30 %). Notably, the gold coating did not affect the ascospore viability.

These results suggest that the ascospores survive different specimen preparation techniques, such as rapid freezing, freeze-drying, and chemical fixation, and imaging conditions, including electron beam radiation and vacuum. However, hyphae, fungal vegetative cells, did not survive any of the treatments (Read and Lord 1991). Therefore, ascospores were assumed to be in a state of suspended animation during SEM observation. Furthermore, despite resuming normal growth after cryo-SEM observations, the ‘living’ ascospores did not ‘grow’ during SEM observations, as commented by Kaminskyj (2021).

### Tardigrades surviving SEM vacuum

Tardigrades, also referred to as water bears, are microscopic eight-legged invertebrates. Due to their remarkable cryptobiosis capabilities over a broad range of environmental extremes, tardigrades are recognized as one of the most indestructible organisms on Earth. These minute metazoans are found in diverse habitats including the deep sea, rivers, mountains, tropical rain forests, and deserts (Persson et al. 2011). However, most tardigrades contract into a quiescent tun state to survive desiccation (Hibshman et al. 2020).

**Fig. 4:**
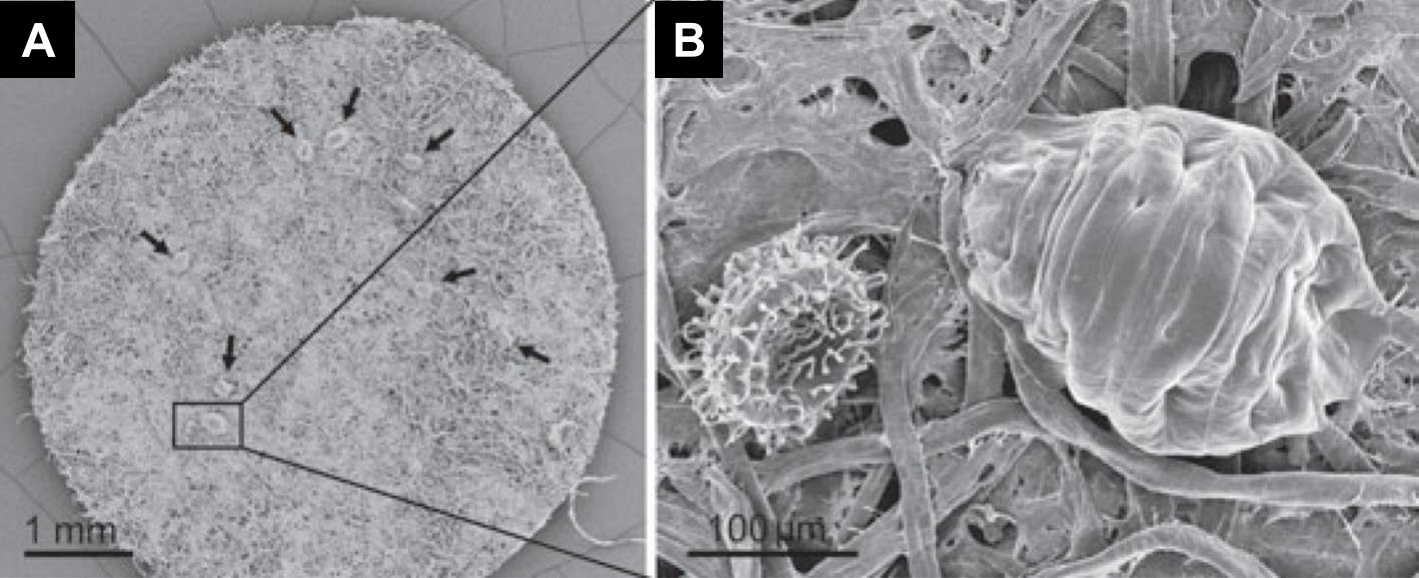
Scanning electron micrographs of the tardigrade *Richtersius coronifer*. **A** Overview showing tardigrades (arrows) on a filter paper. **B** Magnified view of a rectangle in (A) showing an egg (left) and a tardigrade (right) (Persson et al. 2011 with permission from the publisher)

An experiment was performed to test whether the tardigrade *Richtersius coronifer* could survive vacuum (Persson et al. 2011). The animals were placed on desiccated Whatman-3 filter paper mounted on a stub and placed in the SEM observation chamber for 24 h. The vacuum pressure was approximately 2.9 × 10^−8^ Pa. The specimens showed a shrunken morphology (Fig. 4 A and B). After vacuum exposure, the animals were rehydrated and tested for their survival. All animals (20 specimens with three replications) survived the SEM vacuum condition. Despite the lack of an evident effect of vacuum exposure on initial survival, long-term survival after vacuum exposure was several days shorter than that displayed by animals not exposed to vacuum (Persson et al. 2011). Tardigrade-specific intrinsically discharged proteins are essential for desiccation tolerance as these form non-crystalline amorphous solids upon desiccation (Boothby et al. 2017).

### Ticks surviving uncoated SEM observation

Nymphs and adults of a wild tick (*Haemaphysalis flava*) were mounted on stubs and directly observed using SEM (1.5 × 10^−3^ Pa) at 2-5 kV without chemical fixation and metal coating (Ishigaki et al. 2012). The ticks were scanned in the TV mode for 30 min. After SEM observation, they were transferred to a test tube and maintained at room temperature to observe their body motions.

**Fig. 5:**
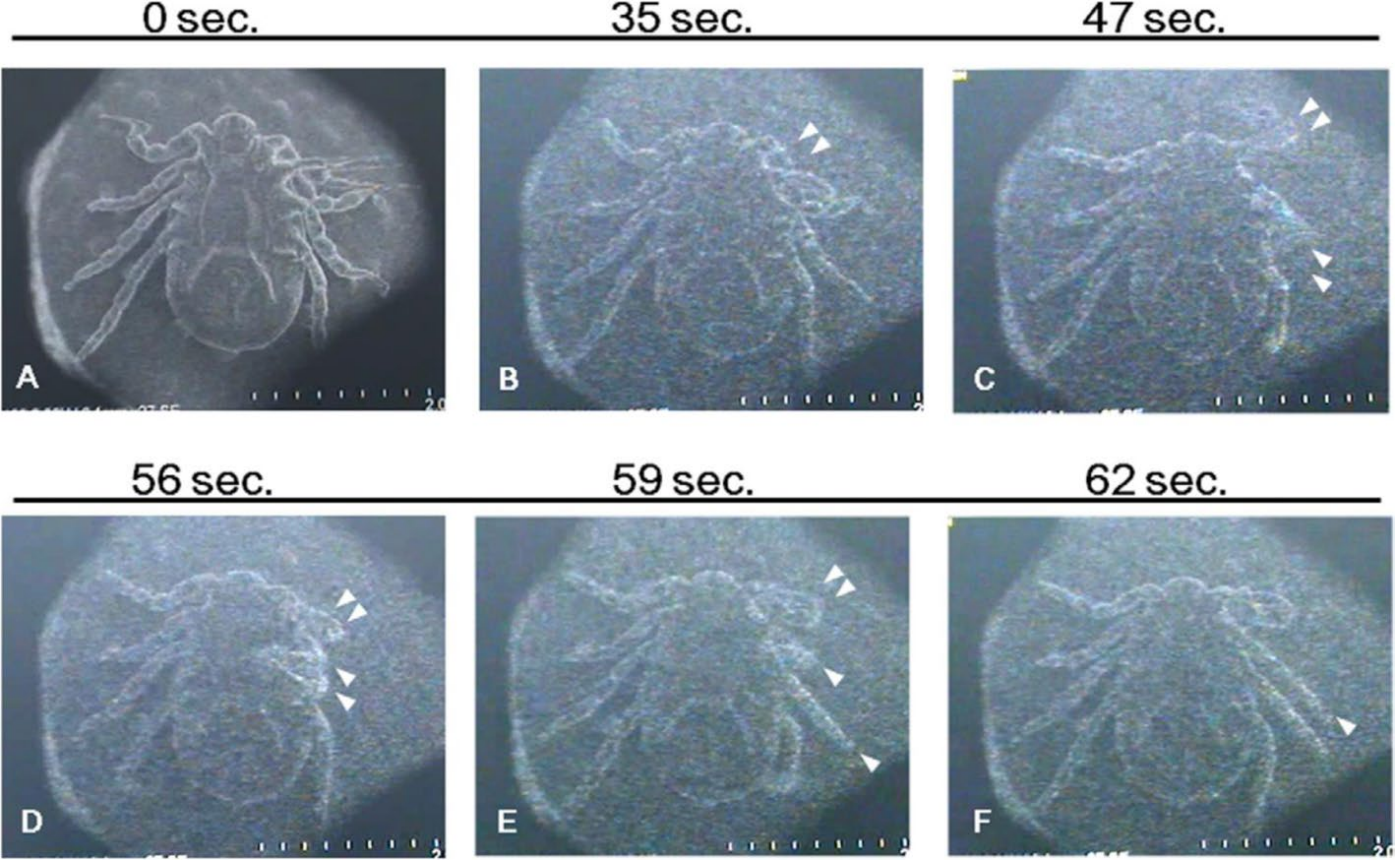
Scanning electron micrographs of a tick *Haemaphysalis flava*. **A-F** The motion of a live tick. Time under vacuum conditions is shown on the top of each micrograph. Arrowhead = leg movement (From Ishigaki et al. 2012 with permission from the publisher)

The specimens actively moved all four legs during TV mode scanning (Fig. 5) (Ishigaki et al. 2012). Later, the ticks stopped moving during SEM observation; however, they resumed their growth after being removed from the SEM. Meanwhile, ticks exposed only to vacuum without an electron beam survived more than 2 weeks; however, the specimens exposed to vacuum and electron beam died in 2 days (Ishigaki et al. 2012). These results suggest that the ticks are resistant to vacuum, but sensitive to electron beam radiation. Furthermore, the mechanism of vacuum resistance might be associated with the breath-stopping function as shown in insects (Ishigaki et al. 2012).

### Mosquito larvae surviving electron beam irradiation

The concept of nano-suit was proposed and applied for imaging of living multicellular organisms (Takaku et al. 2013). A simple surface-rendering method produces a thin polymer layer, referred to as a nano-suit, on the specimens, protecting them against SEM observations. A nano-suit can be made through (i) natural extracellular substances (ECSs), (ii) artificial ECSs by electron beam or plasma irradiation, and (iii) artificial ECSs by immersion in 1 % Tween 20 solution for 1 min before electron beam or plasma irradiation.

**Fig. 6:**
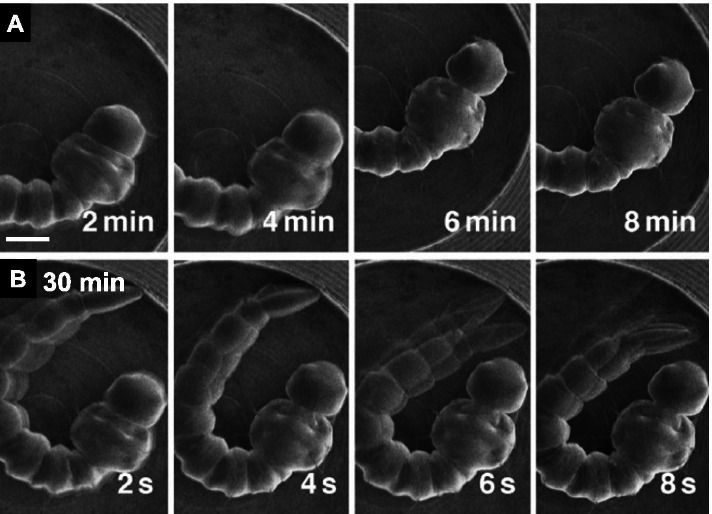
Scanning electron micrographs of the larvae of the Asian tiger mosquito *Aedes albopictus*. **A** The larva immersed in 1 % Tween 20 solution for 1 min before observation. Bar = 0.5 mm. **B** Sequential micrographs of the larva of (**A**) 30 min after observation. Blurred images in (**A**) and (**B**) indicate active movements (Takaku et al. 2013 with permission from the publisher)

Larvae of the Asian tiger mosquito *Aedes albopictus* were immersed in 1 % Tween 20 solution for 1 min and directly observed using SEM at 5 kV for more than 30 min (Takaku et al. 2013). These larvae have a cuticle with no evident ECS on the surface. The specimens retained their morphology and exhibited movements in the observation chamber maintained at the vacuum level of 10^−5^-10^−7^ Pa (Fig. 6 A). They showed movements after 30 min and approximately 80 % of larvae (*n* = 25) developed into pupae and adults (Fig. 6B) (Takaku et al. 2013).

### Midge larvae surviving plasma irradiation

**Fig. 7:**
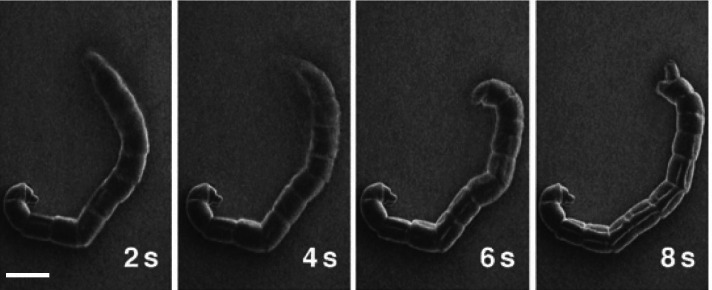
Sequential scanning electron micrographs of the larva of the midge *Chironomus yoshimatsui*. The larva was immersed in 1 % Tween 20 solution for 1 min and irradiated with plasma for 3 min before observation. Bar = 0.3 mm. Blurred images indicate active movements (Takaku et al. 2013 with permission from the publisher)

A midge, *Chironomus yoshimatsui*, possesses a cuticle with no evident ECS on the surface. Its larvae were immersed in 1 % Tween 20 solution for 1 min, irradiated with plasma, and observed using SEM at 5 kV for more than 5 min. The specimens showed well-preserved morphology and active movements during SEM observations (Fig. 7) (Takaku et al. 2013). These results suggest that if the specimens without ECS are covered with a nano-suit through plasma irradiation, they could survive the SEM conditions.

### Using low-energy beam, fast vacuum, and minimal preparations for observing live hydrated specimens

A technique was developed to observe live hydrated specimens using a low-energy electron beam, a rapid vacuum system, and minimal specimen preparations (Scharf 2017). First, insects and spiders were anesthetized via refrigeration and mounted on a stub using denatured alcohol and graphite. Next, they were observed using SEM with a fast pump-down vacuum system at 5 kV. It took approximately 1 min to reach 6.5 × 10^−3^ Pa. After SEM observations, a few specimens were still alive and returned to the field.

## Summary and outlook

The major drawback of conventional EM is the loss of biological activity after observation due to the specimen damage caused by high-energy electron beam radiation and high-vacuum conditions. By using a relatively low-energy electron beam, radiation damage on biological specimens can be reduced during SEM imaging. Insects observed using SEM without chemical fixation were the first organisms that were reported to be alive after SEM observation. Thereafter, other eukaryotes belonging to the taxa of plants, arachnids, animals, and fungi were reported to survive SEM conditions, such as vacuum and electron beam radiation. The surviving organisms were observed (i) without chemical fixation, (ii) after mounting on a precooled cold stage, (iii) using cryo-SEM, or (iv) after coating with a thin polymer layer (Table 1). Combined use of these techniques may provide a better condition for preservation and live imaging of multicellular organisms for a long time beyond live-cell EM. Artificial coating with a thin polymer layer was effective for active movements of organisms during and after SEM observations, expanding the scope of the specimen taxa. Living multicellular eukaryotes after high-resolution SEM observations could offer a better platform for unveiling biological processes.

**Table 1:** Eukaryotes surviving scanning electron microscopy (SEM) conditions

Organisms	SEM conditions	Survival criteria	References
Beetle	No chemical fixation	Metamorphosis	Pease et al. 1966
Plant	Cold stage (-15℃)	Protoplasmic streaming	Kaneko et al. 1985
Fungus	Chemical fixation Cryo-fixation	Spore germination	Read and Lord 1991
Tardigrade	No chemical fixation	Movement	Persson et al. 2011
Tick	No chemical fixation	Movement	Ishigaki et al. 2012
Mosquito	No chemical fixation (nano-suit)	Metamorphosis	Takaku et al. 2013
Midge	No chemical fixation (nano-suit)	Movement	Takaku et al. 2013
Insect Spider	No chemical fixation	Movement	Scharf 2017
